# The Immunogenicity of Monovalent Oral Poliovirus Vaccine Type 1 (mOPV1) and Inactivated Poliovirus Vaccine (IPV) in the EPI Schedule of India

**DOI:** 10.3390/vaccines12040424

**Published:** 2024-04-17

**Authors:** Lalitendu Mohanty, T. Jacob John, Shailesh D. Pawar, Padmasani Venkat Ramanan, Sharad Agarkhedkar, Pradeep Haldar

**Affiliations:** 1 Panacea Biotec Ltd., New Delhi 110044, India; 2 Department of Clinical Virology, Christian Medical College, Vellore 632002, India; tjacobjohn@yahoo.co.in; 3 National Institute of Virology, Pune 411001, India; shaileshpawarniv@gmail.com; 4 Department of Paediatrics, Sri Ramachandra Hospital, Chennai 600116, India; padmasani2001@yahoo.com; 5 Department of Paediatrics, Padmashree Dr D.Y. Patil Medical College, Pune 411018, India; agarkhedkar@gmail.com; 6 Ministry of Health and Family Welfare, Government of India, New Delhi 110011, India; pradeephaldar@yahoo.co.in

**Keywords:** poliomyelitis, bivalent oral poliovirus vaccine, monovalent oral type 2 poliovirus vaccine, inactivated poliovirus vaccine, routine immunization, India

## Abstract

Background: In 2016, the Global Polio Eradication Initiative (GPEI) recommended the cessation of using type 2 oral poliovirus vaccine (OPV) and OPV, with countries having to switch from the trivalent to bivalent OPV (bOPV) with the addition of inactivated poliovirus vaccine (IPV) in their routine immunization schedule. The current GPEI strategy 2022–2026 includes a bOPV cessation plan and a switch to IPV alone or a combination of vaccine schedules in the future. The focus of our study was to evaluate the immunogenicity of monovalent OPV type 1 (mOPV1) with IPV and IPV-only schedules. Methods: This was a three-arm, multi-center randomized–controlled trial conducted in 2016–2017 in India. Participants, at birth, were randomly assigned to the bOPV-IPV (Arm A) or mOPV1-IPV (Arm B) or IPV (Arm C) schedules. Serum specimens collected at birth and at 14, 18, and 22 weeks old were analyzed with a standard microneutralization assay for all the three poliovirus serotypes. Results: The results of 598 participants were analyzed. The type 1 cumulative seroconversion rates four weeks after the completion of the schedule at 18 weeks were 99.5% (97.0–99.9), 100.0% (97.9–100.0), and 96.0% (92.0–98.1) in Arms A (4bOPV + IPV), B (4mOPV1 + IPV), and C (3IPV), respectively. Type 2 and type 3 seroconversions at 18 weeks were 80.0% (73.7–85.1), 76.9% (70.3–82.4); 93.2% (88.5–96.1), 100.0% (98.0–100.0); and 81.9% (75.6–86.8), 99.4% (96.9–99.9), respectively, in the three arms. Conclusions: This study shows the high efficacy of different polio vaccines for serotype 1 in all three schedules. The type 1 seroconversion rate of mOPV1 is non-inferior to bOPV. All the vaccines provide high type-specific immunogenicity. The program can adopt the use of different vaccines or schedules depending on the epidemiology from time to time.

## 1. Introduction

Since the launch of the Global Polio Eradication Initiative (GPEI) in 1988, five of the six World Health Organization (WHO) regions have been certified as wild poliovirus-free [1]. The last case of WPV2 was detected in 1999, with the global certification of eradication in 2015 [2]; the last case of WPV3 was detected in 2012, and WPV3 was certified as eradicated in 2019 [3]. Only type 1 wild poliovirus (WPV1) remains, and occupies only two endemic countries—Afghanistan and Pakistan [4]. Both Pakistan and Afghanistan had reported six cases of WPV1 in 2023 [5,6]. No cases of WPV1 have been reported in Nigeria since 2016 [7]. However, the virus was imported into Mozambique from Pakistan, causing eight WPV1 cases to be reported in 2022 [8].

The GPEI Endgame Strategic Plan (2022–2026) aims to sustain a polio-free world by increasing the access to vaccines and transitioning towards government ownership [9]. The Sabin oral poliovirus vaccine (OPV) contains attenuated live poliovirus, which risks mutation and reversion to virulence [10]; thus, OPV withdrawal will eliminate the risk of circulating vaccine-derived poliovirus (cVDPV). Also, few children are affected due to vaccine-associated paralytic polio (VAPP). All OPV will be ultimately stopped and replaced with inactivated poliovirus vaccine (IPV) [11]. The type 2 component of trivalent OPV (tOPV) causes >90% VDPVs, and nearly 40% of VAPP cases [12]. The first milestone of the Endgame Strategic Plan was a globally synchronized switch from tOPV to bivalent OPV (bOPV containing types 1 and 3 Sabin poliovirus), which was implemented in April 2016, with the withdrawal of the type 2 component from the immunization program [13]. To mitigate the risk of type 2 immunity, IPV was gradually introduced in routine immunization (RI) at or after 6 weeks of age in OPV-using countries [13]. IPV, unlike OPV, does not replicate in the gut; therefore, it induces lower levels of intestinal immunity. However, most importantly, IPV administered to children primed with OPV is known to provide a significant boosting effect in mucosal immunity to all poliovirus serotypes [14,15]. Thus, adding IPV to the bOPV schedule could supplement recipients with the immunity for types 1 and 3, while providing a good type 2 baseline immunity to guard against cVDPV2. Since WPV3 has also been eradicated, it open the possibility of removing the type 3 component from bOPV in the future. This could prompt a switch from bOPV to mOPV1—which is based on a similar rationale as the switch from tOPV to bOPV. This will reduce the burden of cVDPVs and VAPPs emerging from the type 3 vaccine virus as well. Even if the program does not consider another switch, higher mOPV1 immunogenicity may justify its use in one form or another to stop or minimize WPV1 or VDPV1.

India, a highly populated country, contributed to over 60% of all global polio cases until 2009. The country was certified as polio-free in 2014, as the last case was reported in 2011 [16]. However, India’s neighboring countries, Afghanistan and Pakistan, continue to report the circulation of WPV1 and cVDPVs [5,6]. Therefore, it is essential to maintain very high population immunity through continued polio vaccinations in India, which will reduce the risk of cVDPVs as well.

The EPI schedule in India includes four doses of bOPV at birth, 6, 10, and 14 weeks, combined with IPV. The introduction of IPV in India moved through a series of changes: the first one comprised full-dose administration at 14 weeks, then two fractional doses at 6 and 14 weeks, and, with the latest revision, three fractional doses of IPV at 6, 14 weeks, and 9 months.

This study in India provides immunological data on bOPV-IPV and its substitution with mOPV1-IPV in RI. Moreover, it could provide support moving forward in the planned steps of the Endgame Strategy, subsequent to the tOPV–bOPV switch. The primary objectives of this study were to compare immunogenicity against poliovirus type 1 with bOPV and mOPV1 in the routine EPI schedule along with a dose of IPV at week 14 of age, and to assess the immunity provided by three doses of standalone IPV in the EPI schedule of India. The secondary objectives of this study included the following: (1) an assessment of gains in immunity for poliovirus types 2 and 3 with one dose of IPV at DTP3 contact, (2) seroconversion for poliovirus types 2 and 3 with two doses of IPV added to mOPV1 schedule, (3) seroconversion or gains in titer of poliovirus-neutralizing antibodies with a booster dose of IPV at 18 weeks, and (4) interference and immunogenicity of pentavalent antigens (DTwP-HepB-Hib) when administered concomitantly with bOPV, mOPV1, and IPV in the EPI schedule. Additionally, the safety of these vaccines was also assessed in this study.

## 2. Methods

This was a three-arm, multi-center, open label, randomized controlled trial undertaken between May 2016 and January 2017 at three medical institutions in India. The study protocol followed good clinical practice standards; ethical clearance was obtained from the WHO Ethical Review Committee at Geneva and by the Institutional Review Board (IRB) of Indian Council of Medical Research and the participating institutions in India. The trial was approved for implementation by the Drugs Controller General-India (DCGI) and registered with the Clinical Trial Registry of India, with the number CTRI/2016/04/006826.

### 2.1. Enrolment of Study Participants

The parents or the legally acceptable representative (LAR) of the study participants underwent an informed consent process, including pre-natal consent for the cord blood collection as well as a written consent for study enrolment if the eligibility criteria were met. The eligibility criteria included a full-term pregnancy, ≥2.5 kg birth weight, ≥9 APGAR score at 5 min, and residence at an accessible distance from the hospital (≤30 km). A difficult labor or postpartum complication, a suspected medical condition or congenital defect, immunodeficiency and thrombocytopenia led to exclusion from the study. The participants were enrolled in the study from birth till 22 weeks after birth. Only the study participants who presented with a complete set of data, as per the protocol analysis, were included in the final analysis.

### 2.2. Study Design

The infants born in the selected medical institutions were enrolled within 24 h of birth into one of the study arms: A (bOPV + IPV), B (mOPV1 + IPV) or C (IPV only). The study design is summarized in Table 1. The children who were assigned to the EPI schedule (Arm A) received doses of bOPV at birth, 6, 10, and 14 weeks. The study participants assigned to Arm B received mOPV1 at birth, 6, 10 and 14 weeks. The children in Arm C received three doses of IPV at 6, 10, and 14 weeks. In addition, the children in all arms also received a dose of IPV at 18 weeks. All the study arms were administered with pentavalent vaccine (DTP-HepB-Hib) at 6, 10 and 14 weeks in addition to the study-specific polio vaccines.

The infants were randomly allocated to the study arms using a centrally labeled, stratified, computer-generated, permuted block randomization (with blocks of sizes 2, 4 or 6) allocated equally to all the three arms. The investigator allocated participants to study arms as per the randomization envelope prepared by the statistician from the sponsoring institution. The random sequence was generated using SAS 9.4 [17]. Neither the parent nor the investigator had any discretion to opt for a particular study arm. Immediately after birth, under sterile conditions, 3.0 mL cord blood was collected from the placental side of the umbilical cord for routine hospital tests and to test for polio antibodies. After informed consent had been given by the parents and the confirmation of the eligibility criteria of the infant, the infant was vaccinated by a trained nurse, as per the study arm. The study participants attended follow-up visits at 6, 10, 14 and 22 weeks to undergo the outlined vaccination/blood withdrawal program. The adverse and severe adverse events were recorded in all the three arms.

Sample Processing and Analysis: The serum specimens from the study participants were stored in a deep freezer (≤20 °C) and were transported in cold-chain to the laboratory for analysis at the ICMR National Institute of Virology (NIV), Mumbai, and the Pune Unit, Maharashtra, India. The serum specimens were tested using the standard microneutralization assay [18] for the detection of neutralizing antibodies against all the three poliovirus types. The poliovirus types 1 and 3 microneutralization assay was performed at NIV Mumbai unit, while type 2 microneutralization was done at NIV, Pune, India. In addition, serum specimens were also tested for antibodies and components of the pentavalent vaccine at Panacea Biotec Ltd.’s Drug Discovery Research (DDR) laboratory at Mohali.

Seropositivity was defined as the presence of neutralizing antibodies, with a reciprocal titer of ≥8. For children with detectable antibodies, seroconversion was defined as a four-fold increase over the expected decline in maternal antibodies (half-life assumed to be 28 days) at that time-point [19]. If there was a change in the antibody titer, from non-detectable (reciprocal titer < 8) to detectable titer (≥8), it was also considered as seroconversion. The analysis was performed separately for the serotypes 1, 2 and 3.

### 2.3. Administered Vaccines

The bOPV and IPV vaccines used in this trial were taken from market lots of vaccines manufactured by Panacea Biotec Ltd. (New Delhi, India) and Sanofi Pasteur Pvt. Ltd. (Navi Mumbai, India), respectively. The mOPV1 vaccines were specifically manufactured by Panacea Biotec Ltd. (New Delhi, India) for the purpose of this trial. Monovalent Oral Poliovirus Vaccine Type-1 (mOPV1) contains a live attenuated Sabin poliovirus strain, which was grown in a primary monkey kidney cell culture. This vaccine contains Sabin poliovirus at no less than 10^6.0^ CCID_50_ per dose. This vaccine mimics the immune response following infection with wild poliovirus Type-1. mOPV1 has been licensed for use in India since 2005. The bivalent type 1 and 3 oral poliovirus vaccine (bOPV) contained at least 10^6.0^ CCID_50_ of Sabin poliovirus type 1 and at least 10^5.8^ CCID_50_ of Sabin poliovirus type 3. The Inactivated Poliovirus Vaccine (IPV) was formulated to contain 40-8-32 D-antigen potency. The conventional dose of bOPV and mOPV1 is 2 drops given orally and 0.5 mL of IPV given intramuscularly. EasyFive-TT is a WHO pre-qualified pentavalent vaccine containing diphtheria, whole-cell pertussis, tetanus, Hib and hepatitis B antigens. EasyFive-TT was administered as a concomitant vaccine through the intramuscular route. 

### 2.4. Statistical Analysis

A WHO collaborative study conducted during 2013–2014 in India showed 99% seroconversion for poliovirus type 1 in the group that received bOPV with IPV at 14 weeks [15]. Assuming cumulative seroconversion of 99%, with an equivalence margin of 5%, power of 99%, and alpha level of 1%, the sample size was calculated to be 172 in each study arm. Considering 20% dropouts and attrition due to some other reasons, it was decided to study 200 children in each of the study arms, making a total of 600 children across all the three study sites.

Differences in the percentage points of the cumulative seroconversion with 95% confidence limits were obtained for poliovirus type 1 only. Per-protocol analysis was used for the analysis of the outcomes. Children whose antibody titers were above or equal to 1448 (highest detectable titer) at the baseline were excluded.

The distribution of demographic variables and titers at baseline were summarized in all the three study arms, by providing the frequencies with the percentages for categorical demographic variables, while the median titers have been presented using bootstrap [5] with 95% CI obtained from 10,000 bootstrap samples for all the three serotypes. The comparison of seroconversion across the arms was performed using Chi-square test and the comparison of doses across the arms was undertaken using the Cochrane–Armitage test. A *p* value < 0.05 was considered to be statistically significant. R 3.4.3 and SAS 9.4 were used for analyzing the data.

## 3. Results

**Figure 1:**
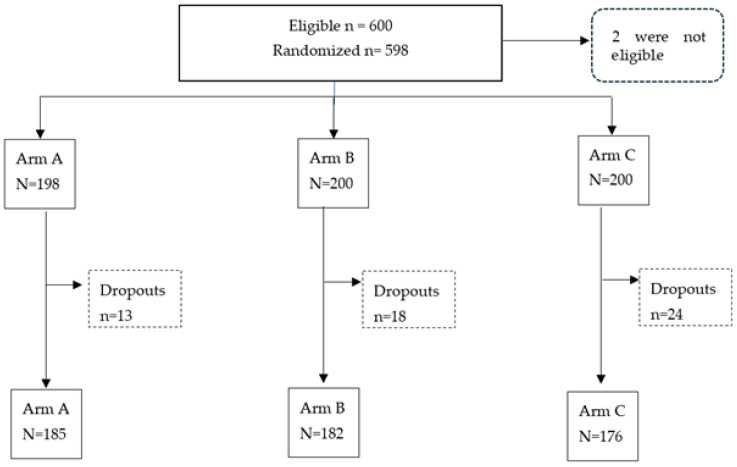
Consort flowchart.

A total of 600 participants were enrolled, as shown in the consort flowchart (Figure 1). In two cases, cord blood could not be collected or the amount was insufficient for testing. Therefore, 598 study participants were subjected to randomization in the study. With an 8.7% drop-out rate, the study was completed with 185 participants in Arm A, 182 participants in Arm B and 176 participants in Arm C.

### 3.1. Baseline Characteristics

The baseline characteristics of the study participants are provided in Table 2. The socio-demographic variables, such as religion and father’s educational level, were comparable across the three arms. There were slightly more female children in Arm A and a similar proportion in the other two study arms. The baseline seroprevalence levels and median titers were not significantly different across the study arms in all the three serotypes.

### 3.2. Seroconversion

Seroconversion values with 95% confidence limits, expressed as a percentage, are presented in Table 3. The cumulative seroconversion rates at 14 weeks in Arms A and B (having received three doses of bOPV and mOPV1, respectively) were 96.8% (93.1–98.5) and 98.4% (95–3-99.4) for type 1 poliovirus (PV) (*p* = 0.323). At 18 weeks, after four doses of the respective vaccine and one IPV dose, seroconversion was 99.5% (97.0–99.9) and 100% (97.9–100) for Arm A and B, respectively. The percentage point difference in seroconversion between Arms A and B (Arm B–Arm A) for serotype 1 was 0.5% (−0.51–1.5), indicating that Arm B was equivalent to Arm A with an equivalence margin of 5%. For type 3 poliovirus, the seroconversion rates at 18 weeks were 100% (98.0–100.0) in Arm A and 81.9% (75.6–86.8) in Arm B (*p* < 0.001).

When the infants received one dose of IPV at week 14 contact, the type 2 seroconversion increased in all three arms: Arm A—21.6% (16.3–28.1) at 14 weeks (3bOPV) to 80.0% (73.7–85.1) (4bOPV + IPV) at 18 weeks (*p* value < 0.001); Arm B—22.0% (16.6–28.5) at 14 weeks (3mOPV1) to 76.9 (70.3–82.4) (4mOPV1 + IPV) at 18 weeks (*p* value < 0.001); Arm C—69.3% (62.1–75.6) (2IPV) to 93.2% (88.5–96.1) (3IPV) for serotype 2 (*p* value < 0.001).

The IPV stand-alone schedule (Arm C) at 14 weeks, having received two doses of IPV, showed 84.7% (78.6–89.4) seroconversion against type 1 poliovirus. At 18 weeks, this seroconversion rate significantly increased to 96.0% (92.0–98.1) with an additional dose of IPV (*p* < 0.01). Type 2 seroconversion also increased significantly from 14 to 18 weeks (69.3% (62.1–75.6) vs. 93.2% (88.5–96.1), (*p* < 0.001)). Finally, seroconversion against type 3 poliovirus was already high at 14 weeks with 94.3% (89.9–96.9); the third dose boosted seroconversion even further to 99.4% (96.9–99.9) (*p* value = 0.01). At 22 weeks, the only IPV schedule (Arm C) received four doses of IPV. The seroconversion rates for the three serotypes with four doses of IPV at week 22 were 97.2% (93.5–98.9), 96.6% (92.8–98.4) and 100% (97.9–100), for serotypes 1, 2 and 3, respectively. The seroconversion rates at 18 weeks were not statistically significant in any of the three serotypes compared to week 22 (*p* value = 0.771, 0.226 and 1.000, respectively).

**Figure 2:**
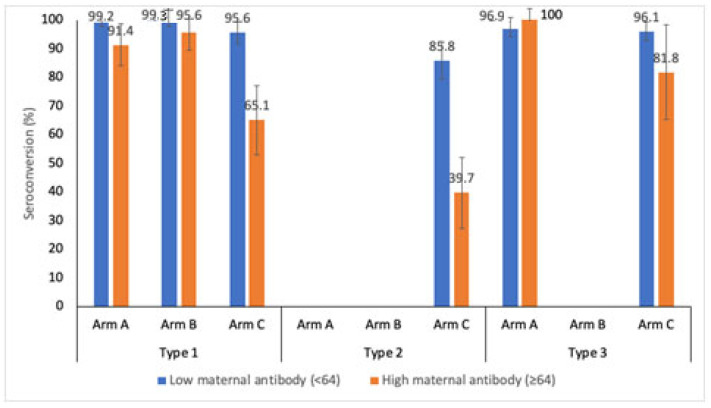
Seroconversion at 14 weeks of age for poliovirus protection with regard to maternal antibody levels. Arm A participants had received 3 bOPV doses, Arm B 3 mOPV1 doses and Arm C 2 IPV doses. The maternal antibodies were measured through the baseline antibody titers.

The immunogenic response against the level of maternal antibodies (baseline titers) was analyzed for the bOPV, mOPV1 and IPV arms at week 14. The maternal antibody levels were categorized as low (<64) and high (≥64). Figure 2 shows the seroconversion for the three poliovirus serotypes stratified by the level of maternal antibodies. Serotype 1 seroconversion at week 14 in Arm A, after three doses of bOPV, was 99.2% (95.7–99.9) and 91.4% (81.4–96.3) for low and high maternal antibody levels, respectively (*p* value = 0.006). Serotype seroconversion rates after administering three doses of mOPV1 in Arm A were 99.3% (96.0–99.9) and 95.6% (85.2–98.8) for low and high maternal antibody levels, respectively (*p* value = 0.088). In the IPV stand-alone arm, the serotype 1 seroconversion rates were 95.6% (90.1–98.1) and 65.1% (52.8–75.7) (*p* value < 0.001) in the low and high maternal antibody strata, respectively. The type 2 component was present only in Arm C (IPV schedule). The seroconversion rates against type 2 poliovirus were found to be 85.8% (78.2–91.1) and 39.7% (28.5–52.0) for low and high maternal antibodies, respectively (*p* value < 0.001). For type 3 poliovirus, the seroconversion rates were 96.9% (92.9–98.7) and 100% (7.4–20.2) (*p* value = 0.382) in the bOPV schedule (Arm A), and 96.1% (91.8–98.2) and 81.8% (61.5–92.7) in the IPV schedule (Arm C), for low and high maternal antibodies, respectively (*p* value = 0.007).

### 3.3. Median Titers

As seen in Table 4, at 14 weeks, the children who received either bOPV or mOPV1 had high median titers as compared to those children who received IPV only (Arm C) (*p* < 0.001 for both). Type 1 median titers increased from 724 at 14 weeks to 1149 at 18 weeks in Arms A and B, while the titers increased from 91 to 144 in Arm C (*p* value< 0.001 for both). At 22 weeks, children who received mOPV1 (Arm B) had higher titers as compared to arms A and C (1149 vs. 912 vs. 228, *p* value < 0.01 for B vs. C and *p* = 0.084 for A vs. B).

Type 2 median titers increased from 45 with two doses of IPV at 14 weeks to 72 with three doses of IPV at 18 weeks in Arm C (*p* value < 0.001). An additional dose of IPV further increased the median titer levels of type 2 poliovirus to 114 (*p* value < 0.001). Arms A and B were seronegative at week 14 (7 (95% CI: 6–7) versus week 6 (95% CI: 6–7)); after the addition of one IPV dose, the median titers increased in both arms to 18 and 23, respectively, at week 18. After an additional IPV dose, the titers increased even further to 57 (95% CI: 51–72) and 72 (95% CI: 57–72) in Arms A and B, respectively.

With three doses of bOPV in Arm A, the median titer for type 3 poliovirus was 287 (95% CI: 228–362) at 14 weeks. With an additional dose of bOPV, it increased significantly to 724 (95% CI: 574–781; *p* value < 0.001) at 18 weeks. The increase in median titer was not significant at 22 weeks (*p* = 0.225). In Arm C, there was a significant increase in median titers across the visits at weeks 14, 18 and 22 from 228 (181–287) to 287 (228–362) to 362 (287–410) (*p* value < 0.001), respectively.

### 3.4. Immune Response of Concomitant EasyFive TT

The immune responses of the component antigens of EasyFive TT were tested by ELISA. The antibody thresholds for seroprotection are ≥0.1 IU/mL, ≥20 U/mL, ≥0.1 IU/mL, ≥0.15 microgram/mL and ≥10 mIU/mL for diphtheria, pertussis, tetanus, *Hemophilus influenza* type b, and hepatitis B, respectively. The seroprotection for all the components of EasyFive TT at 18 weeks were 93.3% (90.7–95.2), 58.8% (54.7–62.9), 99.5% (98.3–99.9), 99.5% (98.3–99.9) and 97.8% (93.7–99.5) for diphtheria, pertussis, tetanus, *Hemophilus influenza* type b, and hepatitis B, respectively. The post-vaccination Geometric Mean Titers (GMTs) were higher than pre-vaccination GMTs for all antigens, as shown in Table 5.

### 3.5. Adverse Events in the Study

A total of 486 adverse events were observed in the study, of which 371 were solicited adverse events and 115 were unsolicited adverse events. The solicited adverse events mainly included 108 events of fever and 142 events of pain at injection site; 83 upper respiratory tract infection (URTI) events and 10 events of acute gastroenteritis were the main unsolicited adverse events in the study.

A total of 11 serious adverse events (SAEs) was observed in the study participants, including seven males and four females. Criteria for SAE was hospitalization in 10 participants and death in one participant. Eight of these participants had respiratory infection. None of the SAEs were causally associated with study vaccines.

## 4. Discussion

There is enough evidence from the literature that bOPV or mOPV1 are more immunogenic than tOPV [20] for the respective serotypes. mOPV1 has been found to be more immunogenic than bOPV for type 1 in some studies, but this difference was not statistically significant in other studies [21]. Our trial also demonstrated that the effects of three doses of mOPV1 were similar to those of three doses of bOPV against type 1 poliovirus. Moreover, the median antibody titer for serotype 1 was equivalent for both bOPV and mOPV1 recipients (724 (574–818) vs. 724 (724–724), *p* = 0.203). This demonstrates that mOPV1 is just as efficacious as bOPV in conferring type 1 poliovirus immunity, as observed in another study [21].

After three doses, IPV showed a 96% seroconversion rate, compared to 98.4% for mOPV1 and 96.8% for bOPV for serotype 1. This shows the comparable effect of all three vaccines in conferring type 1 immunity for the same number of doses. Types 1 and 3 median antibody titers increased to 1149 and 724, respectively, in bOPV recipients. Type 1 seroconversion was raised to 100% and median antibody titers to a maximum of 1149 in mOPV1 recipients.

The addition of one dose of IPV to the bOPV (Arm A) and mOPV1 (Arm B) schedules provided 60–70% seroconversion to type 2, which increased to 97% with the addition of the second dose. IPV also closes the immunity gap against types 1 and 3, as demonstrated in some previous studies [22,23].

### 4.1. Immunogenicity of IPV Schedule

Three doses of the IPV schedule at 6, 10 and 14 weeks provided high immunogenicity of ≥90% against all serotypes. Similar findings were reported in trials from Cuba and the Philippines, with three doses of IPV administered at 6, 10 and 14 weeks of age. The Cuba trial showed a minimum 95% seroconversion rate against types 1 and 3, while the Philippines trial demonstrated 99% seroconversion rates against all the three types [24]. The addition of another dose of IPV closed the immunity gaps further for all the three serotypes. Moreover, at the close of the study, IPV recipients demonstrated high levels of median antibody titers in all the poliovirus (PV) serotypes (PV1: 228 (181–269), PV2: 114 (91–144) and PV3: 362 (287–410)).

The timing of vaccine administration is important due to the interference with maternal antibodies. High levels of maternal antibodies (baseline titers >64) versus low levels showed an especially significant detrimental effect on seroconversion rates in the IPV study arm (95.6% vs. 65.1% for type 1, 85.8% vs. 39.7% for type 2 and 96.1% and 81.8% for type 3). This supports the rationale of vaccinating a child with IPV as late as possible after birth to account for maternal antibody waning. In fact, various studies have shown that a delayed administration in a 2-, 4- and 6-month schedule is more immunogenic than a 6-, 10- and 14-week schedule [25,26].

### 4.2. Concomitant Vaccines

The administration of concomitant vaccines did not interfere with OPV/IPV immunogenicity in this trial.

Our trial has confirmed that bOPV and mOPV1 are highly immunogenic against the respective serotypes. The addition of IPV fills the immunity gap effectively for the remaining types, and boosts the effects of OPV. An IPV-only schedule in early age should be a three- or four-dose schedule, and the same applies to a combination vaccine like wP- based hexavalent vaccine.

Since WPV3 has been globally eradicated and WPV1 still persists, replacing bOPV with mOPV1 could be one of the options for the program. The results of this study provide a basis for future GPEI strategies regarding the withdrawal of all oral poliovirus vaccines and the continuation of IPV or combination vaccines in routine vaccination.

### 4.3. Limitations

The major limitation of the present study is that unexpected type 2 seroconversion was observed in Arms A and B at 14 weeks, even though no type 2 poliovirus-containing vaccine was received by the participants until that time. Some inadvertent exposure to type 2-containing poliovirus vaccines or undetected type 2 vaccine-derived poliovirus cannot be ruled out, but both seem very unlikely. There was a very limited availability of IPV in India during the study implementation phase, and the absence of detection of poliovirus type 2 from case and environmental surveillance was very unlikely given the sensitive surveillance in the country. We tried to investigate the reasons for the unexplained type 2 seroconversion, but no specific reasons could be elucidated. Additionally, type 2 neutralization had to be conducted in different facilities; however, the testing protocols were similar for all three serotypes. Also, at the time of publication, we could not retrieve the data on all ANC mothers who were approached for cord blood collection in the study. Therefore, this figure could not be added to the consort diagram. The study was not designed based on any representative sample, as it was a clinical trial with study participants taken from the population of the catchment area pertaining to the study sites (medical institutes).

## 5. Conclusions

This study allows us to build confidence in the steps required in the eradication of polio, subsequent to the tOPV–bOPV switch. This study shows the high efficacy of all proposed vaccine schedules in evoking high seroconversion rates and median antibody titers. Thus, each arm can be considered for use in the next phases of the polio eradication strategy: bOPV + IPV in the post-switch era, mOPV1 + IPV for WPV1 eradication and IPV alone after the certification of WPV eradication. VDPVs could be dealt with via novel oral poliovirus vaccines with or without IPV in outbreak response.

## Figures and Tables

**Table 1:** Trial design.

Study Arms		Arm A (bOPV + IPV)	Arm B (mOPV1 + IPV)	Arm C (IPV Only)
**Study Visit Schedule**		**Study Vaccines and Samples**		
**Birth**	Sample	Cord blood	Cord blood	Cord blood
	Vaccine	bOPV	mOPV1	__
6 * weeks	Sample	__	__	__
	Vaccine	bOPV	mOPV1	IPV
10 * weeks	Sample	__	__	__
	Vaccine	bOPV	mOPV1	IPV
14 * weeks	Sample	Blood	Blood	Blood
	Vaccine	bOPV + IPV	mOPV1 + IPV	IPV
**18 weeks**	Sample	Blood	Blood	Blood
	Vaccine	IPV	IPV	IPV
**22 weeks**	Sample	Blood	Blood	Blood

* At 6, 10 and 14 weeks, the pentavalent vaccine (DTP-HepB-Hib) was given in all the study arms, in addition to the study-specific polio vaccines.

**Table 2:** Distribution of the baseline characteristics for the study population.

Characteristics	Arm A N = 198		Arm B N = 200		Arm C N = 200	
	n	%	n	%	n	%
**Female child vs. male child**	104	52.5	87	43.5	93	46.7
**Hindu religion vs. other religion**	181	91.4	175	87.5	176	88.0
**Father’s education**						
Illiterate	8	4.0	12	6.1	10	5.0
Primary/Middle	62	31.3	55	27.9	53	26.6
High school/Higher secondary	84	42.4	89	45.2	86	43.2
Graduate/Higher	44	22.2	41	20.8	50	25.1
**Normal weeks of gestational vs. not normal**	198	100.0	200	100.0	200	100.0
**Normal Apgar score vs. abnormal Apgar score**	198	100.0	200	100.0	200	100.0
**Baseline Seroprevalence**						
Type 1	172	86.9	167	83.5	174	87.0
Type 2	182	91.9	168	84.0	174	87.0
Type 3	130	65.7	126	63.0	123	61.5
**Baseline Titers**						
**Type 1**						
Median	29		29		29	
(95% CI)	(23–45)		(23–36)		(23–36)	
**Type 2**						
Median	36		36		45	
(95% CI)	(36–45)		(29–45)		(36–45)	
**Type 3**						
Median	14		11		11	
(95% CI)	(11–18)		(10–14)		(9–14)	

**Table 3:** Cumulative seroconversion rates in all study arms.

	Arm A* (N = 185)			Arm B** (N = 182)			Arm C*** (N = 176)		
	n	%	95% CI	n	%	95% CI	n	%	95% CI
**14 weeks**									
**Type 1**	179	96.8	93.1–98.5	179	98.4	95.3-99.4	149	84.7	78.6–89.4
**Type 2**	40	21.6	16.3–28.1	40	22.0	16.6–28.5	122	69.3	62.1–75.6
**Type 3**	180	97.3	93.8–98.8	43	23.6	18.0–30.3	166	94.3	89.9–96.9
**18 weeks**									
**Type 1**	184	99.5	97.0–99.9	182	100.0	97.9–100.0	169	96.0	92.0–98.1
**Type 2**	148	80.0	73.7–85.1	140	76.9	70.3–82.4	164	93.2	88.5–96.1
**Type 3**	185	100.0	98.0–100.0	149	81.9	75.6–86.8	175	99.4	96.9–99.9
**22 weeks**									
**Type 1**	185	100.0	98.0–100.0	182	100.0	97.9–100.0	171	97.2	93.5–98.9
**Type 2**	181	97.8	94.6–99.2	176	96.7	93.0–98.5	170	96.6	92.8–98.4
**Type 3**	185	100.0	98.0–100.0	180	98.9	96.1–99.7	176	100.0	97.9–100.0
	**Type 1**			**Type 2**			**Type 3**		
	**A vs. B**	**A vs. C**	**B vs. C**	**A vs. B**	**A vs. C**	**B vs. C**	**A vs. B**	**A vs. C**	**B vs. C**
**14 weeks**	0.323	<0.001	<0.001					0.162	
**18 weeks**	0.824	0.024	0.006	0.522	<0.001	<0.001	<0.001	0.891	<0.001
**22 weeks**		0.022	0.023	0.525	0.495	0.959	0.153		0.729

Arm A*: 14 weeks—3 bOPV; 18 weeks—4bOPV + IPV; 22 weeks—4bOPV + 2IPV. Arm B**: 14 weeks—3 mOPV1; 18 weeks—4mOPV1 + IPV; 22 weeks—4mOPV1 + 2IPV. Arm C***: 14 weeks—2IPV; 18 weeks—3 IPV; 22 weeks—4 IPV.

**Table 4:** Median titers.

Titers	Arm A				Arm B				Arm C			
	Median		95% CI		Median		95% CI		Median		95% CI	
**14 weeks**												
**Type 1**	724		(574–818)		724		(724–724)		91		(91–114)	
**Type 2**	7		(6–7)		6		(6–7)		45		(36–57)	
**Type 3**	287		(228–362)		6		(6–6)		228		(181–287)	
**18 weeks**												
**Type 1**	1149		(912–≥ 1448)		1149		(1149–1152)		144		(114–181)	
**Type 2**	18		(16–23)		23		(18–29)		72		(57–91)	
**Type 3**	724		(574–781)		36		(23–57)		287		(228–362)	
**22 weeks**												
**Type 1**	912		(724–1149)		1149		(912–1176)		228		(181–269)	
**Type 2**	57		(51–72)		72		(57–72)		114		(91–144)	
**Type 3**	724		(724–724)		287		(228–362)		362		(287–410)	
	**Type 1**				**Type 2**				**Type 3**			
	**A vs. B**	**A vs. C**		**B vs. C**	**A vs. B**	**A vs. C**		**B vs. C**	**A vs. B**	**A vs. C**		**B vs. C**
**14 weeks**	0.203	<0.001		<0.001	0.654	<0.001		<0.001	<0.001	0.006		<0.001
**18 weeks**	1.000	<0.001		<0.001	0.778	<0.001		<0.001	<0.001	<0.001		<0.001
**22 weeks**	0.084	<0.001		<0.001	0.876	<0.001		<0.001	<0.001	<0.001		0.103

Arm A: 14 weeks—3 bOPV; 18 weeks—4bOPV + IPV; 22 weeks—4bOPV + 2IPV. Arm B: 14 weeks—3 mOPV1; 18 weeks—4mOPV1 + IPV; 22 weeks—4mOPV1 + 2IPV. Arm C: 14 weeks—2IPV; 18 weeks—3 IPV; 22 weeks—4 IPV.

**Table 5:** Pre- and post-vaccination GMTs of component antigens of EasyFive-TT.

Antibody	GMT	
	Pre-Vaccination	Post Vaccination
Anti-diphtheria (IU/mL)	0.093 (0.086–0.102)	0.471 (0.435–0.513)
Anti-tetanus (IU/mL)	1.58 (1.123–2.051)	2.184 (1.87–2.55)
Anti-PRP (microgram/mL)	0.64 (0.592–0.690)	2.91 (2.59–3.26)
Anti-HBs Ag (mIU/mL)	0.00088 (0.001–0.001)	471.31 (438.61–506.46)
Anti-Pertussis IgG (U/mL)	2.89 (2.30–4.06)	16.54 (13.55–20.86)

## Data Availability

Data are unavailable due to privacy or ethical restrictions.
